# Retracted: Further Increase in the Expression of Activation Markers on Monocyte-Derived Dendritic Cells in Coronary Artery Disease Patients with Ectasia Compared to Patients with Coronary Artery Disease Alone

**DOI:** 10.1155/2020/4641013

**Published:** 2020-11-02

**Authors:** Mediators of Inflammation

*Mediators of Inflammation* has retracted the article titled “Further Increase in the Expression of Activation Markers on Monocyte-Derived Dendritic Cells in Coronary Artery Disease Patients with Ectasia Compared to Patients with Coronary Artery Disease Alone” [1]. This article has been retracted at the request of the authors.
